# Implementation of an Online Drug–Drug Interaction Screener for the STRIVE Ensitrelvir Trial for COVID-19

**DOI:** 10.1093/ofid/ofaf327

**Published:** 2025-06-11

**Authors:** Joshua P Havens, Nayon Kang, Lucy Chung, Courtney V Fletcher, Page Crew, Jacqueline Nordwall, Lianne Siegel, Katrina Harper, Birgit Grund, Marcelo Losso, Shikha Vasudeva, Kyle C Molina, Adit A Ginde, Ryosuke Shimizu, Ahmad Mourad, Alpha Diallo, Mina Pak, Anne Davis-Karim, Phiona Nabaggala, Alfredo J Mena Lora, Derek W Russell, Sho Saito, Jason V Baker

**Affiliations:** University of Nebraska Medical Center, College of Medicine, Department of Infectious Diseases, Omaha, Nebraska, USA; Medical Science and Computing/Guidehouse in Support of National Institute of Allergy and Infectious Diseases; CAMRIS International (Under Contract No. 75N93019D00025 with National Institute of Allergy and Infectious Diseases, NIH, DHHS); University of Nebraska Medical Center, College of Pharmacy, Department of Pharmacy Practice, Omaha, Nebraska, USA; National Institute of Allergy and Infectious Diseases, National Institute of Health, Bethesda, Maryland, USA; University of Minnesota, School of Public Health, Division of Biostatistics and Health Data Science, Minneapolis, Minnesota, USA; University of Minnesota, School of Public Health, Division of Biostatistics and Health Data Science, Minneapolis, Minnesota, USA; University of Minnesota, School of Public Health, Division of Biostatistics and Health Data Science, Minneapolis, Minnesota, USA; University of Minnesota, College of Liberal Arts, School of Statistics, Minneapolis, Minnesota, USA; CICAL and Hospital J.M. Ramos Mejia, Emergent Diseases Research Unit, Buenos Aires, Argentina; Veterans Administration Medical Center, Salem, Virginia, USA; University of Colorado, School of Medicine, Department of Emergency Medicine, Aurora, Colorado, USA; University of Colorado, School of Medicine, Department of Emergency Medicine, Aurora, Colorado, USA; Clinical Pharmacology and Pharmacokinetics, Shionogi & CO, Ltd., Osaka, Japan; Division of Infectious Diseases, Department of Medicine, Duke University School of Medicine, Durham, North Carolina, USA; Duke Clinical Research Institute, Durham, North Carolina, USA; ANRS Emerging Infectious Diseases, Clinical Research Safety Department, Paris, France; University of Texas Southwestern Medical Center, Department of Pharmacy Services, Dallas, Texas, USA; Veterans Affairs Cooperative Studies Program Pharmacy Coordinating Center, Albuquerque, New Mexico, USA; Medical Research Council/Uganda Virus Research Institute & London School of Hygiene and Tropical Medicine, Uganda Research Unit, Entebbe, Uganda; University of Illinois at Chicago, Division of Infectious Diseases, Chicago, Illinois, USA; The University of Alabama at Birmingham, Division of Pulmonary, Allergy, & Critical Care Medicine, School of Medicine, Birmingham, Alabama, USA; Veterans Administration Medical Center, Birmingham, Alabama, USA; National Center for Global Health and Medicine, Disease Control and Prevention Center, Tokyo, Japan; Hennepin Healthcare, Department of Infectious Diseases, Minneapolis, Minnesota, USA

**Keywords:** COVID-19, clinical trial, drug–drug interaction, ensitrelvir, online trial resource

## Abstract

**Background:**

Ensitrelvir is an antiviral agent against severe acute respiratory syndrome coronavirus 2 (SARS-CoV-2) with associated drug–drug interactions (DDIs) through CYP3A, *P*-glycoprotein (*P*-gp), breast cancer resistance protein (BCRP), and organic anion transporter-3 (OAT-3) inhibition. We present the development and implementation of an online DDI screener to assess interactions during the STRIVE ensitrelvir trial.

**Methods:**

The STRIVE Network is conducting a randomized, double-blind, placebo-controlled trial evaluating ensitrelvir's efficacy and safety in hospitalized adults with coronavirus disease 2019 (COVID-19) and lower respiratory tract involvement. DDI guidance was compiled into a database accessed via a web portal where a multidisciplinary team categorized medications as permitted, prohibited, or conditionally permitted. For prohibited medications, washout periods and start/restart criteria were provided with alternative medication suggestions. Sites could request new medications for addition. After 18 months, a survey was conducted to assess the tool's usefulness.

**Results:**

Version 1 of the DDI screener launched in December 2022 with 615 medications, expanding to 1182 through 6 updates by version 7. In 11 cases, prohibited medications were revised to conditionally permit enrollment after dosage adjustments (antihypertensives, anti-infectives, and psychiatric medications). Anticoagulants, immunosuppressants, and emergency use medications posed the greatest challenges due to trial blinding. With 334 participants enrolled across 150 sites in 13 countries, 117192 screener searches were completed by May 2024. The most searched medication classes were antihypertensive, antibiotics, corticosteroids, and anticoagulants. Sites found the DDI screener most helpful during screening/enrollment and valued the washout guidance.

**Conclusions:**

DDI resources for investigational medications like ensitrelvir, with high DDI potential, are crucial for safe conduct of clinical trials. Effective implementation requires a multidisciplinary, iterative approach that incorporates real-time feedback from trial sites.

Since identification of severe acute respiratory syndrome coronavirus 2 (SARS-CoV-2) in 2020, vaccines, treatments, and viral evolution have altered coronavirus disease 2019 (COVID-19) clinical impact [1–5]. Despite this, cases persist, and new treatments are needed for high-risk and hospitalized patients. Early antivirals like molnupiravir (MOV) and nirmatrelvir/ritonavir (NMVr) were mostly studied in the outpatient setting before the emergence of the Omicron SARS-CoV-2 variant (late during 2021), while remdesivir was studied in both the inpatient and outpatient settings [2–4]. Monoclonal antibodies have shown reduced efficacy against newer variants [6]. Two oral antivirals (NMVr and MOV) are available for mild to moderate COVID-19 infection [3, 4], but both have limitations including COVID-19 rebound [7–9], drug–drug interactions (DDIs; NMVr) [10, 11], and lower effectiveness in reducing hospitalizations and deaths (MOV) compared with other antivirals [2–4]. Since the emergence of the Omicron variant, the spectrum of illness for COVID-19 has become less severe, though a subset of participants still progress to require hospitalization (eg, influenced by the presence of comorbidities, older age, or other risk factors). Ensitrelvir, an oral 3CL protease inhibitor approved in Japan [12–15], has demonstrated efficacy against SARS-CoV-2 variants, including Omicron [16–18], with rapid viral load declines [19, 20]. In phase 2/3 trials (SCORPIO-SR and -HR), ensitrelvir demonstrated antiviral activity against Omicron SARS-CoV-2 variants and significantly reduced symptom duration in SCORPIO-SR while showing no evidence of viral rebound in either study [20, 21].

The Strategies and Treatments of Respiratory Infections and Viral Treatments (STRIVE) group is a global network of >200 study sites across 27 countries [22, 23] investigating ensitrelvir (also known as S-217622) among hospitalized patients with COVID-19. The STRIVE ensitrelvir trial is a randomized, double-blind trial assessing the efficacy (primary outcome: days to recovery scale over 60 days of follow-up) and safety of ensitrelvir (375 mg day 1, 125 mg days 2–5) vs placebo (1:1) among hospitalized adults with COVID-19 and evidence of lower respiratory tract involvement (NCT05605093; Supplementary Figure 1) [23]. Like NMVr, ensitrelvir exhibits DDI potential, complicating its use where use of multiple concomitant medications (eg, hospitalized patients) is common. Ensitrelvir is a substrate for cytochrome P450 (CYP) 3A and *P*-glycoprotein (*P*-gp) and an inhibitor of CYP3A, P-gp, breast cancer resistance protein (BCRP), and organic anion transporter-3 (OAT-3) [24, 25]. Importantly, ensitrelvir exhibits a longer elimination half-life (∼48 hours) than NMVr (∼6 hours), presenting risk of ensitrelvir-related DDI for up to 10 days after the last ensitrelvir dose [26]. At the outset of the STRIVE ensitrelvir trial, ensitrelvir DDI guidance resources (eg, University of Liverpool COVID-19 Drug Interaction Checker [27], etc.) were not available. Thus, the management of DDIs posed a significant challenge in the setting of a large international, double-blind trial among hospitalized patients, particularly given the frequent use of corticosteroids and anticoagulants as standard-of-care COVID-19 treatment in this population.

The STRIVE ensitrelvir trial leadership assembled a multidisciplinary team to address DDI guidance/management during the trial and develop DDI resources for trial sites. The primary DDI resource is an online web portal (“DDI screener”) developed specifically for the STRIVE ensitrelvir trial. Here, we present the methods and key constructs for DDI screener development and implementation. Additionally, we present survey results summarizing STRIVE ensitrelvir trial site usage of and initial experiences with the DDI screener.

## METHODS

### DDI Team Development

The STRIVE ensitrelvir trial team assembled an internal team in August 2022 to develop a trial-specific DDI resource. The STRIVE DDI team consists of 4 pharmacists, 2 pharmacologists, 4 physicians, and 3 statisticians meeting on a biweekly basis. Two information technology professionals provide technical support. The STRIVE ensitrelvir trial protocol was finalized in November 2022.

### DDI Guidance Development

Initial DDI resources included the investigator's brochure, protocol instruction manual, discharge communication materials, and a list of commonly used concomitant medications including those for emergency use (eg, amiodarone, diazepam, etc.). For each medication, assessments of substrate sensitivity (mild to strong) for ensitrelvir inhibition pathways (ie, CYP3A, P-gp, BCRP, OAT-3), narrowness of therapeutic index, and half-life were completed. Given the blinded study design, DDI guidance for concomitant medications was developed to account for predicted DDI effects if the participant was assigned to ensitrelvir or placebo. Medications were categorized as permitted, prohibited, or conditionally permitted with dosage restrictions (ie, dose reduction or maximum threshold). DDI studies with other CYP3A inhibitors were evaluated for each medication added to the screener, if available; medications with Cmax and/or area under the curve increases >2 times were generally categorized as prohibited or conditionally permitted with dosage adjustments. For conditionally permitted and prohibited medications, additional guidance was developed, assigning a washout period, start/restart timeline, and supplementary guidance for use.

Washout periods accounted for near full clearance of the concomitant medication before initiation of ensitrelvir and approximated 5 half-lives rounded to the nearest day. Start/restart timelines for medications consisted of 3 time periods dependent on metabolism pathway, substrate sensitivity, narrowness of therapeutic index, and extended ensitrelvir half-life (∼48 hours). Supplementary Table 1 outlines the start/restart timelines. Additional guidance for each medication was included in the “Comments” section, if applicable, for any preferred alternative medications and specific clinical considerations for use with ensitrelvir, particularly for conditionally permitted medications.

The STRIVE DDI pharmacist team members conducted the initial medication review, assigned washout periods, and proposed the categorization for each new drug in the DDI screener. The degree of expected change in plasma concentrations of the concomitant medication and/or ensitrelvir and the toxicity profile of the concomitant medication were important considerations. Recommendations were discussed and finalized by consensus among all STRIVE DDI team members. The proposed screener categorizations for each version were reviewed by Shionogi pharmacologists and the trial sponsor (National Institutes of Health/National Institute of Allergy and Infectious Diseases [NIAID]). If different recommendations were suggested, the STRIVE DDI team members would then resolve conflicts and make final determinations before implementation and presentation to STRIVE sites.

### DDI Screener Implementation

The STRIVE DDI team created an online web portal for trial sites to access the STRIVE DDI screener (https://insight-trials.org/DDI/). Concomitant medications, provided with US-based naming with alternative spelling provided in parentheses (eg, levothyroxine, “levotiroxine”), can be searched on the DDI screener, providing sites with DDI guidance and clinical implications for use with ensitrelvir. If the medication is included in the DDI screener database, the search result returns to a summary guidance table. If there is no potential for DDI, then the search results indicate that use of the given medication is permitted during the trial. If the medication has a potential DDI with ensitrelvir, summary guidance indicates that use of the medication is prohibited and provides the washout period required at screening, dosage adjustment (conditionally permitted medications only), start/restart timeline, and supplementary guidance. If the searched medication is not listed in the DDI screener (entirely or alternative spelling/naming), sites may request addition of the medication to the DDI screener through an embedded link and/or use DrugBank [28] to assess for any potential DDIs with ensitrelvir. Further guidance is provided on the landing page including 2 tables: (1) general guidance on certain medication classes (ie, vitamins/supplements, inhaled/topical medications, etc.) and (2) interacting medications with long half-lives requiring longer washout periods.

Iterative updates to the DDI screener consisted of the DDI team reviewing medications that were requested by sites, key medication classes, and DDI screener format changes based on site feedback. Any medications requested for addition or change of categorization within the DDI screener were evaluated using metabolism pathway information and published DDI data, if available. Conditionally permitted medications were determined on a case-by-case basis depending on the risks and benefits of use with ensitrelvir within the inpatient clinical setting. The DDI team presented all proposed DDI screener additions/changes to the trial safety team, the trial sponsor (NIH/NIAID), and the industry partner (Shionogi) for additional input before each version update. With each version change, a summary of changes and full list of categorized medications is listed on the trial website for sites to use locally (eg, developing DDI alerts within the electronic health record [EHR]).

### Supplemental DDI Resources

The STRIVE DDI team developed complementary DDI resources including a 2-sided quick reference guide (Side A, DDI guidance of the top 100 medications; Side B, emergency use medication guidance), discharge/transition-of-care forms, and participant wristbands (Supplementary Data).

### STRIVE Trial Site Survey

The STRIVE DDI team completed a cross-sectional survey of STRIVE sites to assess their experiences, usage, and perspectives on the DDI screener 18 months after the start of the STRIVE ensitrelvir trial. The survey included 15 questions in 3 parts: (1) trial site demographics; (2) DDI screener perspectives; and (3) DDI screener usage characteristics (Supplementary Data). The survey was voluntary and completed once for each trial site. No respondent identifiers were collected. Eligibility criteria included trial sites that had enrolled ≥1 participant and/or used the DDI screener to screen ≥10 potential participants. The survey was developed in REDCap [29, 30] and disseminated to STRIVE trial sites electronically. All survey data were stored in a MySQL or Oracle database, located on a secure server hosted by the University of Minnesota.

### Data Analysis

Descriptive statistics were used to summarize site demographics, DDI screener perspectives, and DDI screener usage characteristics. Counts and percentages were used for categorical data, and median (range) or mean (SD) was used for continuous data.

### Patient Consent

The University of Minnesota Institutional Review Board (IRB) approved the DDI screener survey protocol and survey (STUDY00021431) with exemption status. All participants provided written informed consent before completion of the survey.

## RESULTS

Version 1 of the DDI screener was officially released for STRIVE use in December 2022, with 615 total medications included. The DDI screener was updated 6 times through its current version (v7), which was implemented in May 2024. As of May 1, 2024, the STRIVE ensitrelvir trial has enrolled 334 participants across 150 open trial sites (Supplementary Figure 2).

### DDI Screener Composition and Metrics

**Figure 1.**
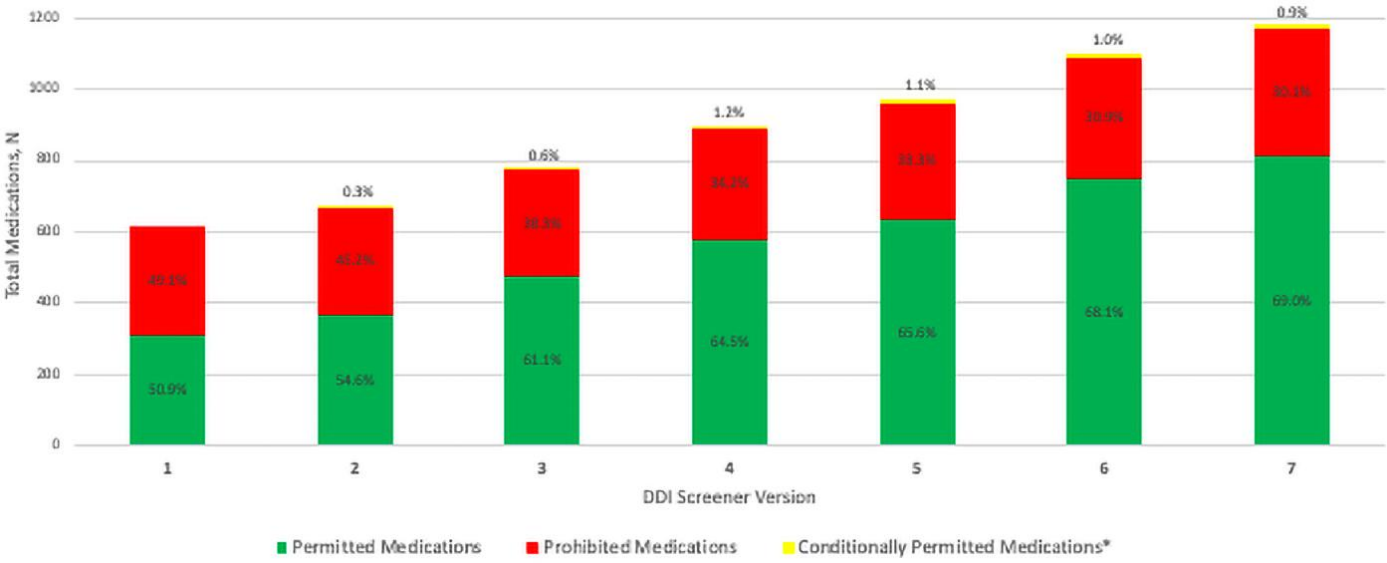
Total medications in the STRIVE DDI screener by version and screener category. Screener category proportions are noted for each version. ^a^Dosage adjustments include dosage reduction or use of maximum thresholds. Abbreviations: DDI, drug–drug interaction; STRIVE, Strategies and Treatments of Respiratory Infections and Viral Treatments.

Through implementation of v7 of the DDI screener, a total of 1182 medications have been added (Figure 1). Version 7 includes 815 permitted (69.0%), 356 prohibited (30.1%), and 11 conditionally permitted (0.9%) medications. The median (range) change in number of medications per DDI screener version is 10.9% (6.8%–19.0%). From v1 to v7, permitted and prohibited medications increased by 160.4% and 17.9%, respectively.

### DDI Screener Updates

Table 1 outlines the changes made to the DDI screener through implementation of v7. The STRIVE DDI team had specific areas of focus (eg, specific medication classes, comparison with other DDI resources as they became available, etc.) with each version update. Of the 603 total changes made to the DDI screener, 550 (91.2%) were medications added to the DDI screener. Fifty-three medication categorization revisions were made; 20 (37.7%) changed from prohibited to permitted, 3 (5.7%) changed from permitted to prohibited, 11 (20.8%) changed to conditionally permitted, and 19 (35.8%) were entry description changes. Dosing guidance for medications changed to conditionally permitted included a maximum dosage threshold (n = 6, 55%) or a 50% dosage reduction (n = 5, 45%). Conditionally permitted medications broadly included anticholesterol (statins), antihypertensive (calcium-channel blockers), anti-infective (antifungal, antibiotic), and antidepressant medications. Most of the DDI guidance revisions resulted from feedback by site investigators, leading to further discussion by the STRIVE DDI team. Generally, DDI guidance revisions were driven by newly provided pharmacokinetic/metabolism information provided to the STRIVE DDI team, particularly with medications not approved in the United States.

**Table 1.** Per Version DDI Screener Additions and Revisions. All Values Noted as No. (%) Unless Otherwise Specified

Screener Version	Total No. of Changes	Total Additions	Total Revisions	Revisions				Version Focus
				Changed to permitted	Changed to prohibited	Changed to conditionally permitted	Entry description changed	
2	44 (7.3)	40	4	0	0	2	2	Antihypertensives, anti-infectives
						Atorvastatin^a^		
						Amlodipine^b^		
3	127 (21.1)	121	6	0	1	3	2	Anticancer, antibiotics, inhaled medications
						Trazodone^a^		
						Sertraline^a^		
						Tamsulosin^a^		
4	139 (23.1)	114	25	17	2	6	0	Comparison with Liverpool resource [22], non-US listed medications
						Methotrexate^c^		
						Clarithromycin^b^		
						Diltiazem^b^		
						Nifedipine^b^		
						Itraconazole^a^		
						Voriconazole^a^		
5	57 (9.5)	55	2	0	0	0	2	Site requested entries, non-US listed medications
6	143 (23.7)	133	10	3	0	0	7	Site requested entries
7	93 (15.4)	87	6	0	0	0	6	Site requested entries
Total	603 (100.0)	550 (91.2)	53 (8.8)	20 (37.7)	3 (5.7)	11 (20.8)	19 (35.8)	…
Median (range)	110.0 (44–143)	100.5 (40–133)	6.0 (2–25)	2.5 (0–17)	0.0 (0–2)	1.0 (0–6)	2.0 (0–7)	…

Abbreviations: DDI, drug–drug interaction; ESV, ensitrelvir.

^a^Conditionally permitted medications with a dosage maximum threshold.

^b^Conditionally permitted medications with dosage reduction guidance.

^c^Conditionally permitted if not dosed during the 5-day ESV treatment course.

### DDI Screener Usage Characteristics

**Figure 2.**
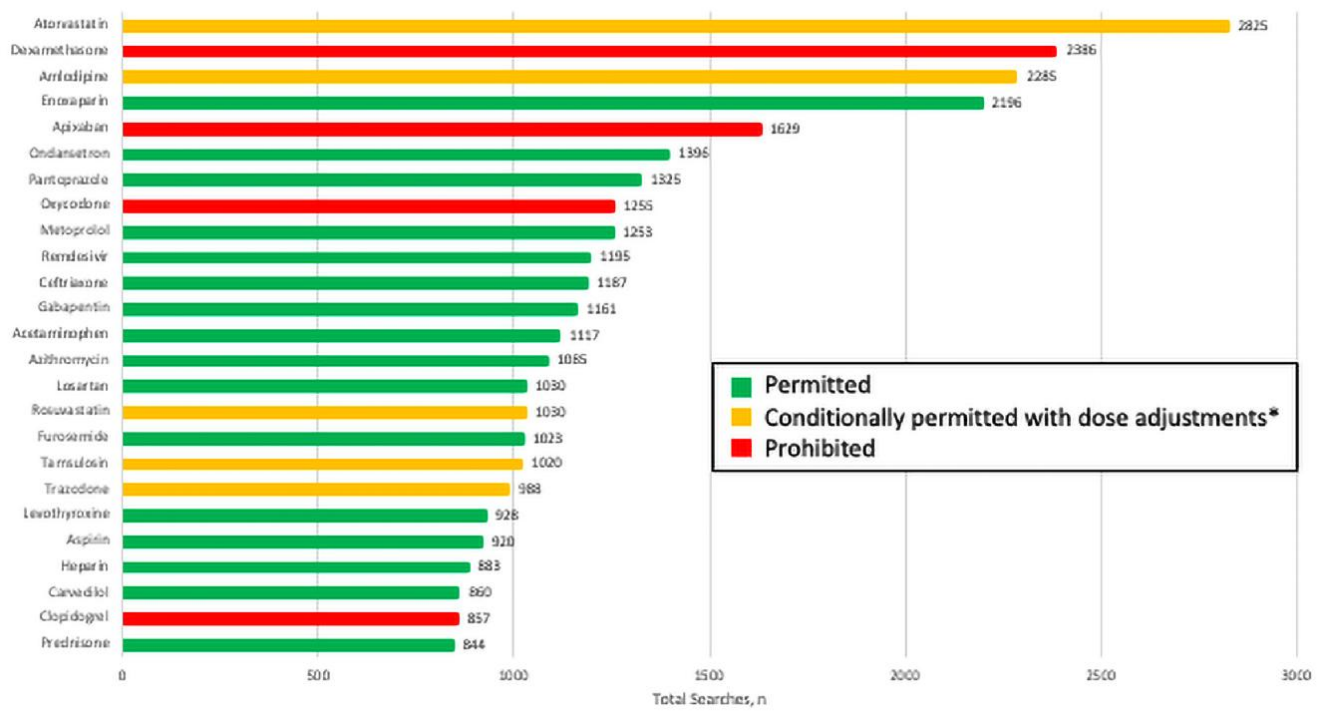
Top 25 medication searches in STRIVE DDI screener by drug name. ^a^Dosage adjustments include reduction or use of maximum thresholds. Abbreviations: DDI, drug–drug interaction; STRIVE, Strategies and Treatments of Respiratory Infections and Viral Treatments.

A total of 117 192 searches have been conducted by STRIVE sites through v7 (Supplementary Figure 3). The mean (SD) number of total searches by DDI screener version was 19 532 (16 453). Figure 2 and Supplementary Figure 4 outline the most searched medications and medication classes, respectively. The most common medication classes searched were antihypertensives (11.6%), antibiotics (7.2%), systemic corticosteroids (5.7%), anticoagulants (5.6%), and anticholesterol (5.0%) medication classes. The 5 most searched medications were atorvastatin (n = 2825, 2.4%), dexamethasone (n = 2386, 2.0%), amlodipine (n = 2285, 1.9%), enoxaparin (n = 2196, 1.9%), and apixaban (n = 1629, 1.4%). Importantly, both atorvastatin and amlodipine were categorized as conditionally permitted in the v2 update (listed as prohibited in v1).

### STRIVE Ensitrelvir Trial Site Survey

**Figure 3.**
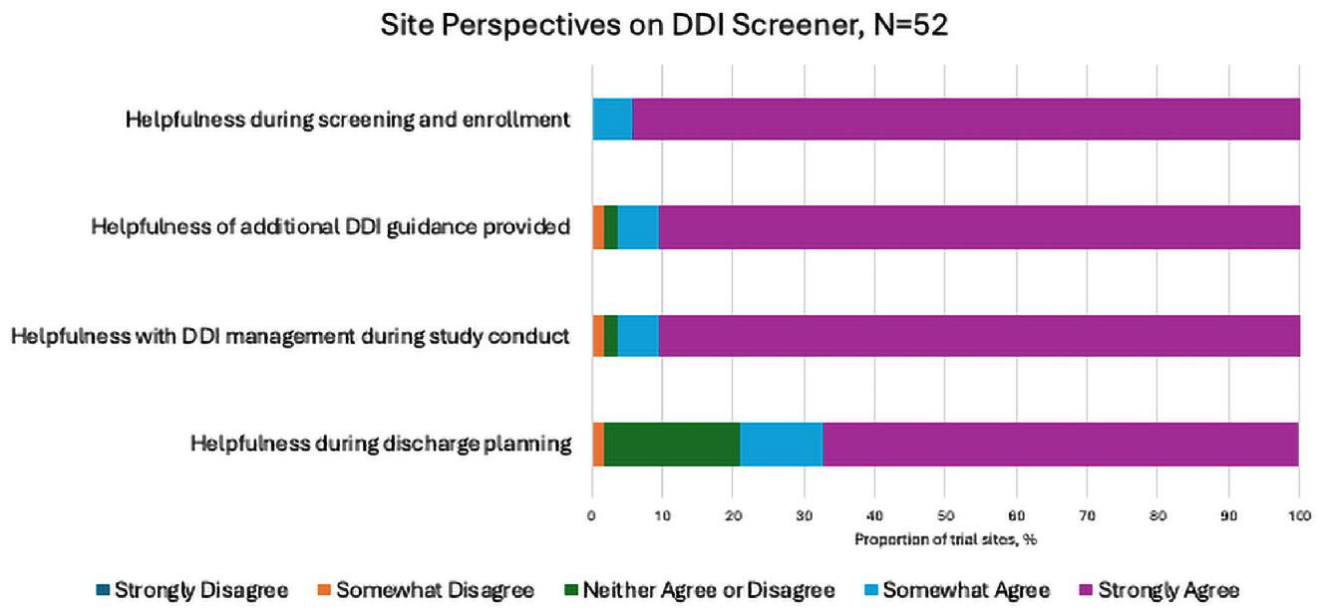
STRIVE trial site perspectives on helpfulness of DDI screener. Abbreviations: DDI, drug–drug interaction; STRIVE, Strategies and Treatments of Respiratory Infections and Viral Treatments.

**Figure 4.**
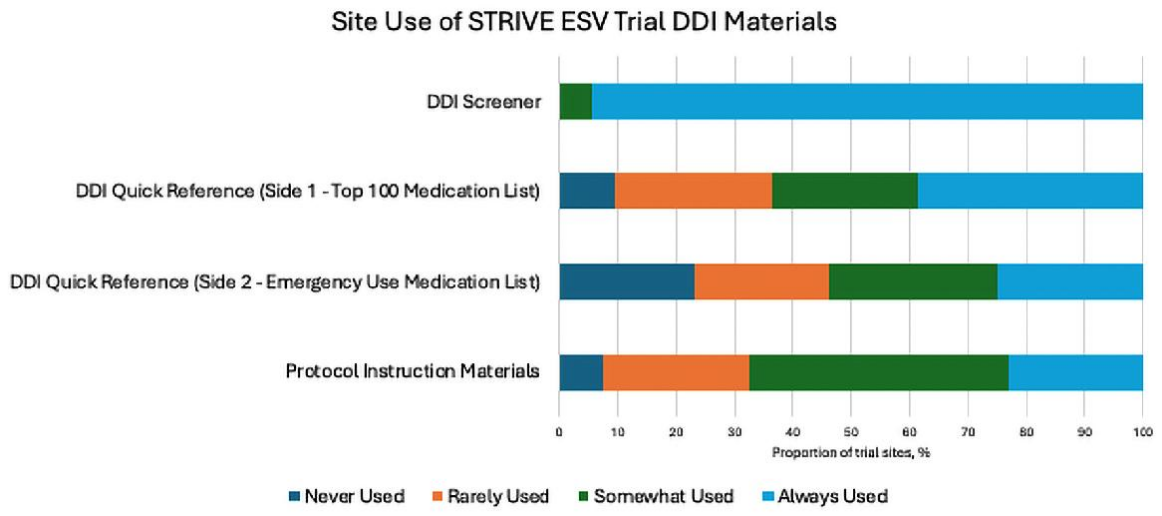
STRIVE trial site frequency of use of trial DDI materials. Abbreviations: DDI, drug–drug interaction; ESV, ensitrelvir; STRIVE, Strategies and Treatments of Respiratory Infections and Viral Treatments.

The DDI screener survey was sent to the sites when ∼290 participants were enrolled, 18 months after enrollment started. Open STRIVE sites were sent the DDI screener survey, with 88 (n = 156, 56.4%) responding. Of these, 73 (83.0%) sites were eligible to complete the survey, with 52 (59.1%) sites enrolling ≥1 participant. Nearly 60% of enrolling sites reported screening >50 participants, and 71% had enrolled 1–5 participants for the STRIVE ensitrelvir trial (Supplementary Table 2). STRIVE sites generally found the DDI screener to be helpful, particularly when establishing eligibility at screening and enrollment (Figure 3). Sites ranked the guidance for the washout period required for concomitant medications during screening/enrollment as the most important feature of the DDI screener (Supplementary Figure 5). The DDI screener was the most used DDI resource, with 94.2% of trial sites reporting that they always used the DDI screener (Figure 4). When a medication was not found in the DDI screener, sites most frequently reported awaiting DDI guidance from the DDI team/trial leadership (50%), utilizing other available DDI resources to guide enrollment/clinical decisions (48%), and/or consulting a clinical pharmacist (48%) (Supplementary Figure 6).

#### Clinical Pharmacist Consultation

Thirty-seven (71.2%) trial sites reported having access to a clinical pharmacist for DDI consultation. Trial sites reported frequent utilization (“very often” or “always”) of clinical pharmacists for DDI consultation during the screening and enrollment period (43%) and when a medication was not found in the DDI screener (39%) (Supplementary Figure 7).

#### Electronic Health Record Integration

Technological integration of the DDI screener within the trial site’s EHR was reported by 44% (n = 23) of STRIVE sites. Twelve (52%) sites embedded the DDI screener hyperlink in the EHR, and 8 (35%) sites developed best practice alerts specifically for ensitrelvir DDI during the order entry process using a list of prohibited medications within the DDI screener, available on the STRIVE website (Supplementary Figure 8).

## DISCUSSION

The STRIVE ensitrelvir trial is a double-blind, placebo-controlled study of a 5-day course of ensitrelvir in hospitalized patients with COVID-19. As a CYP3A, P-gp, BCRP, and OAT-3 inhibitor with a prolonged half-life (∼48 hours), ensitrelvir presents significant challenges for managing DDIs and maintaining patient safety given the study's blinded, placebo-controlled design. The DDI screener, with 1182 medications and >117 000 searches completed through v7, has been instrumental in screening participants and guiding safe DDI management. STRIVE sites generally found the DDI screener to be helpful during all phases of trial conduct. The DDI screener was the most used DDI resource, and most trial sites believed the washout guidance of concomitant medications provided during the screening and enrollment period was the most useful feature.

Constructing DDI guidance for the STRIVE ensitrelvir trial required careful evaluation of the bidirectional interaction potential between ensitrelvir and concomitant medications, especially given ensitrelvir's prolonged half-life. Pharmacokinetic data were critical for determining appropriate washout periods and timing of the concomitant medication initiation during and after the last dose of ensitrelvir. Such guidance, however, is often limited by the availability of pharmacokinetic information, particularly for non–Food and Drug Administration–approved and/or herbal medications, necessitating ongoing, iterative updates as new data emerge.

The blinded, placebo-controlled design of the STRIVE ensitrelvir trial added complexity to DDI management, as conventional DDI resources (eg, University of Liverpool [27], Lexicomp [31], etc.) do not account for blinding. For example, general guidance for apixaban (ie, a substrate of CYP3A4 and *P*-gp with a narrow therapeutic index) with a CYP3A4 inhibitor, such as ensitrelvir, would recommend cautionary use with apixaban dosage reduction (ie, 2.5 mg twice daily, assuming a patient does not already meet apixaban dose reduction criteria) [32]. However, a dosage reduction during the STRIVE ensitrelvir trial could potentially result in underanticoagulation if the participant had been randomized to placebo [12, 27, 31, 32]. While therapeutic drug monitoring (TDM) could mitigate this, the availability and rapidity of concomitant medication blood concentration monitoring are unpredictable [33–36]. Further, TDM will delay dose changes and risk unblinding participants and research/clinical teams, introducing bias.

Similarly, the use of emergency medications with DDI potential (eg, amiodarone, diltiazem, sedatives) poses considerable DDI challenges, particularly in a population of hospitalized patients, such as those in the STRIVE ensitrelvir trial. Decompensating patients requires rapid intervention with emergency medications, sometimes regardless of DDI. In these scenarios, close clinical monitoring for toxicity with careful titration and TDM is imperative to patient safety. Consideration of discontinuation of the trial intervention and/or unblinding is dependent on participant clinical status and potential continued need for any concomitant medications with DDI risk. Thus, medications with narrow therapeutic indexes, such as apixaban, pose the greatest DDI guidance challenges.

Successful implementation of the STRIVE DDI screener required an iterative, multidisciplinary approach as it is inherently an evolving tool requiring continuous review and updating. Input from pharmacologists, pharmacists, clinicians, statisticians, the broader network scientific oversight committee, and other key stakeholders (NIAID, Shionogi, Inc.) was required, in addition to web portal technical support. Ongoing management and improvement of the DDI screener involved a mechanism for site investigators and staff to provide feedback, particularly important within a global trial. Thus, regular communication, discussion, and research calls with sites were vitally important to communicate feedback and present version updates. Still, the iterative approach often results in time gaps between version updates and the addition of medications to the DDI screener. Avenues for DDI guidance of medications not found in the DDI screener, such as pharmacist consultations or providing other DDI resources, were necessary while sites awaited version updates.

The public availability of the DDI screener, along with its complete list of medications, has enabled its ease of use and local customization. The website requires no login, making it accessible to clinical and research teams for evaluating concomitant medications for STRIVE participants. Some STRIVE sites have integrated the DDI screener into EHR tools (Supplementary Figure 8), including embedding the DDI screener hyperlink within the ensitrelvir/placebo order set, prompting assessment of DDIs throughout the follow-up period (screening to study day 29). Other sites have implemented best practice alerts using the DDI screener's list of prohibited and conditionally permitted medications to notify clinicians of potential DDI risks and adjust treatments accordingly.

A DDI screener, such as that presented here, may have drug development value to provide wider applicability for studies investigating medications with multiple significant drug interactions. Rather than excluding all medications for which there was no DDI pharmacokinetic information (as might be more commonly done), the screener development allowed informed judgment as to medications that should be prohibited, could be allowed, or could be allowed with dose adjustment. Therefore, information arising out of the use of the DDI screener could be used by industry and regulatory bodies in formulating broader DDI guidance in clinical settings.

The DDI screener site survey had limitations, particularly responder bias, as responding sites likely had more experience with the DDI screener and potentially stronger opinions. This should be considered when interpreting our findings and their applicability. Further, the DDI screener is intended exclusively for use within the STRIVE study. Following regulatory approval, product use should align with the approved labeling.

## CONCLUSIONS

To our knowledge, the STRIVE ensitrelvir trial DDI screener is the first online, interactive DDI management tool specifically developed for a double-blind, placebo-controlled trial. Implementation required a multidisciplinary and iterative approach to add new data and respond to feedback from clinical sites. Concomitant medication dose adjustments required careful consideration to protect blinding of the study drug assignment. DDI resources for medications like ensitrelvir with high DDI potential are essential for safe conduct of clinical trials. Based on usage data, informal feedback, and site survey data, this online DDI screener met this need for the STRIVE ensitrelvir trial. The development, implementation, and use of this DDI screener provide a roadmap for the development of such a tool for other investigational medications in clinical development.

## Supplementary Material

ofaf327_Supplementary_Data

## Supplementary Data

Supplementary materials are available at *Open Forum Infectious Diseases* online. Consisting of data provided by the authors to benefit the reader, the posted materials are not copyedited and are the sole responsibility of the authors, so questions or comments should be addressed to the corresponding author.
