# Supplementary material for: Implementation of an Online Drug–Drug Interaction Screener for the STRIVE Ensitrelvir Trial for COVID-19
Source: Open Forum Infect Dis. 2025 Jun 11;12(7):ofaf327. doi: 10.1093/ofid/ofaf327 (PMC12207740; doi:10.1093/ofid/ofaf327)
Supplement: ofaf327_Supplementary_Data [file ofaf327_supplementary_data.zip › STRIVE_DDI_Supplementary Appendix.docx]

**Supplementary Appendix**

STRIVE Drug-Drug Interaction Resources

*Quick Reference (Side A) – Common Medications*


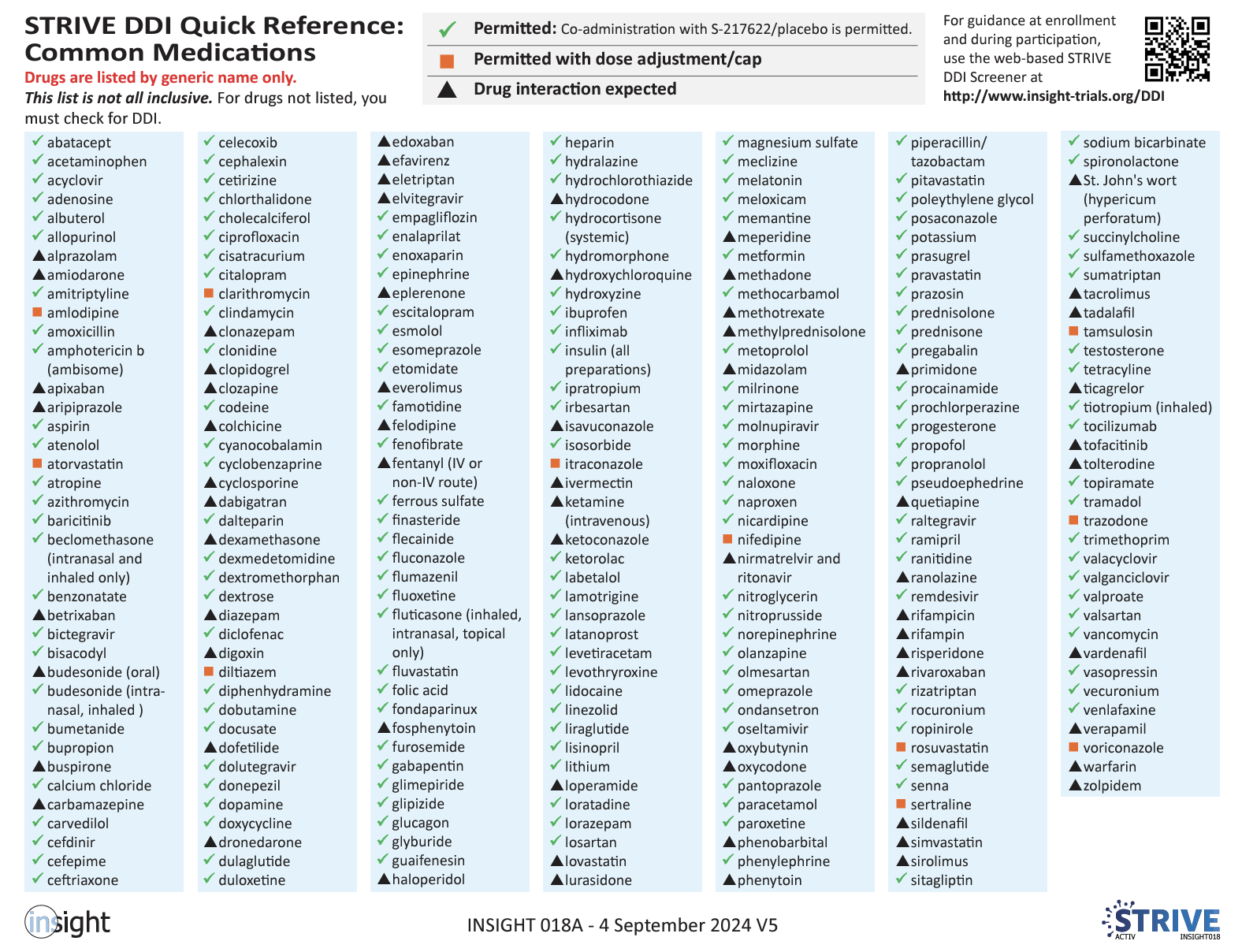


*Quick Reference (Side B) – Emergency Use Medications*


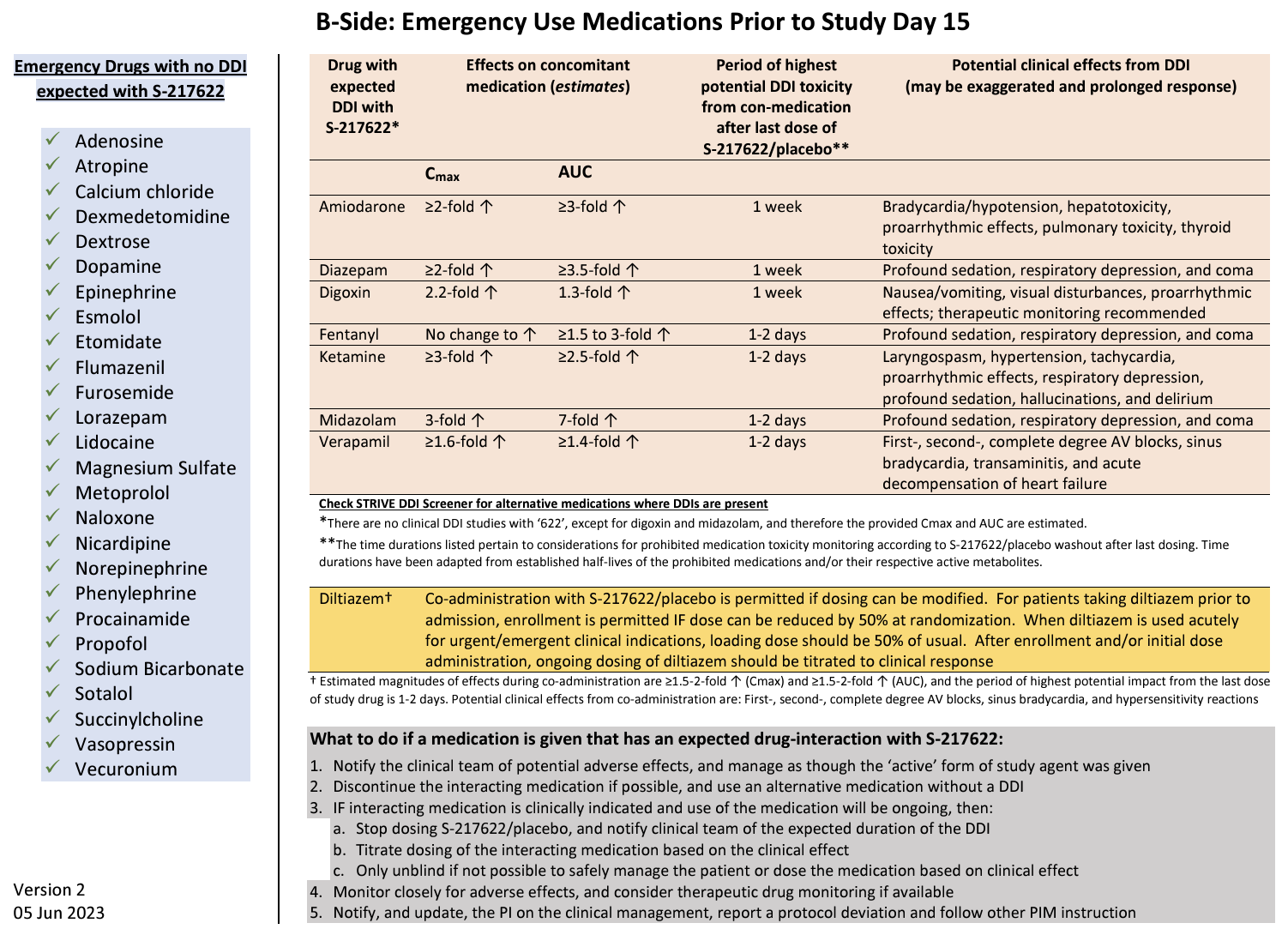


*Concomitant Medication Worksheet*

**
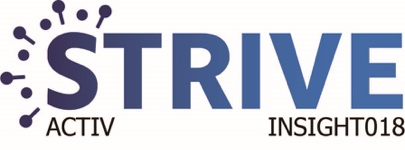
Enrollment Concomitant Medication Reconciliation Worksheet**

Patient Identification Number (PID): ___________

Date of Form Completion: __________

STRIVE DDI Screener Version (http://www.insight-trials.org/DDI/): ___________

| Study Randomization Date (Day 0) |  |
| --- | --- |
| Study Day 5 Date |  |
| Study Day 15 Date |  |
| Study Day 29 Date |  |

| Concomitant Medication | Is Medication Prohibited Due To DDI?  (Yes or No) | Prohibited Medication Start/Restart Timeline | Action Taken Before Enrollment or During Study  (i.e. held or switched) | Prohibited Medication  Restart Date | Notes |
| --- | --- | --- | --- | --- | --- |
|  |  |  |  |  |  |
|  |  |  |  |  |  |
|  |  |  |  |  |  |

**
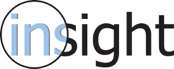
**

**INSIGHT018A V1 January 2023**

*Discharge Wallet Card*


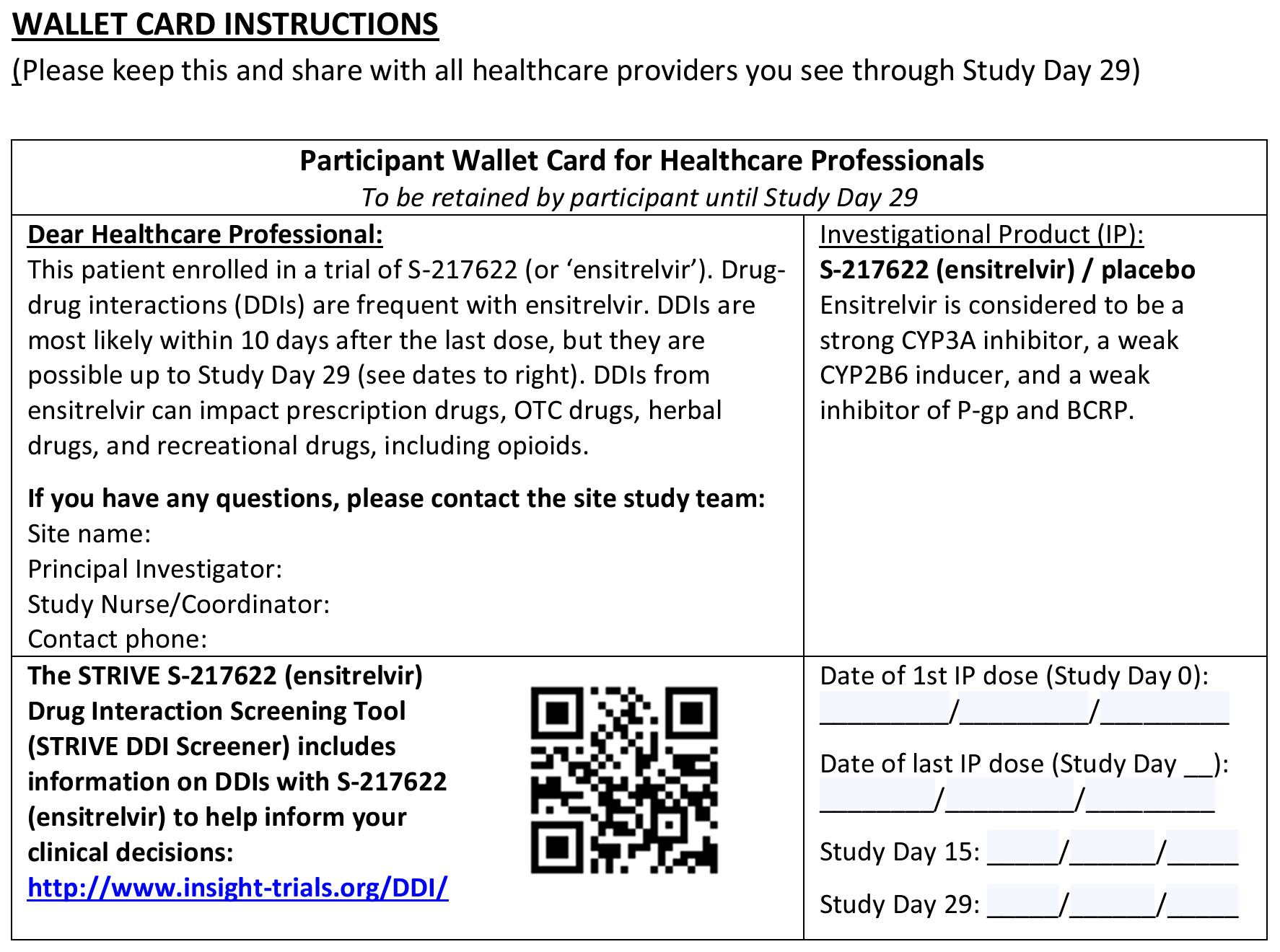


*Discharge Wristbands*

*
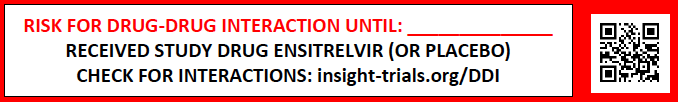
*

STRIVE Trial Site Survey

*STRIVE Ensitrelvir Trial Drug-Drug Interaction Screener Site Survey*

*I*nvitation
You are being invited to participate in this survey study because you are representing an open, study site for the STRIVE E-1 Ensitrelvir trial.

Purpose
The purpose of this study is to collect your beliefs/perspectives of the STRIVE Drug-Drug Interaction (DDI) screener.

Methods

This study includes completion of a single survey. The content of the survey includes 2 sections:

1. Study site demographics (i.e. geographic location, number of participants enrolled, etc)
2. Perspectives of the STRIVE DDI screener.

*Study Directions*

1. **This study aims to collect perspectives of the STRIVE DDI screener from study sites that have enrolled ≥1 study participant and/or have used the screener to screen ≥10 potential study participants.**
2. **The survey should be completed only once by site personnel best suited to address questions involving the site's use of the STRIVE DDI screener (i.e. have used the screener).**
3. **This study is optional and you may choose to opt out.**

*Confirmation of Study Eligibility*

Has your site used the STRIVE DDI screener to screen ≥10 potential study participants *or* enrolled ≥1 participant?

1. Yes, then below:
   1. By checking the box, you consent to completing the STRIVE DDI screener survey.
   2. By checking this box, you or your site team decline participation and do not want to complete this survey.
2. No, then below:
   1. Thank you. Your site is not eligible to complete the study survey

Date:

Site Number:

*Part 1: Site Demographics*

1. Approximately how many STRIVE E-1 participants has your site ***screened*** for STRIVE E-1 using the DDI screener?
   1. 0-9
   2. 10-50
   3. 51-100
   4. 101-500
   5. >500
2. Approximately how many STRIVE E-1 participants has your site ***enrolled*** for STRIVE E-1?
   1. 0
   2. 1-5
   3. 6-10
   4. 11-15
   5. >15
3. Approximately how many total clinical trials does your site’s team participate in annually?
   1. 0-1
   2. 2-3
   3. 4-5
   4. >5
   5. Unsure
4. Does your site have a dedicated pharmacist (research or clinical) available for drug-drug interaction (DDI) consultation?
   1. Yes
   2. No
   3. Unsure

*Part 2: Perspectives on the DDI screener*

In the following questions, we wish to learn about your thoughts and beliefs regarding the online STRIVE drug-drug interaction (DDI) screener.

In Questions 1-4, please mark the answer that best indicates your level of agreement with each statement.

1. The STRIVE DDI screener has been helpful ***during the screening and enrollment*** of potential participants.
   1. Strongly agree
   2. Somewhat agree
   3. Neither agree nor disagree
   4. Somewhat disagree
   5. Strongly disagree
2. The **additional guidance for concomitant medications (i.e. alternative permitted medications, dosage cap thresholds/reductions, etc)** provided by the STRIVE DDI screener has been helpful when screening participants and during trial conduct.
   1. Strongly agree
   2. Somewhat agree
   3. Neither agree nor disagree
   4. Somewhat disagree
   5. Strongly disagree
3. The STRIVE DDI screener has been helpful ***when determining concomitant medication management during study conduct (i.e., for a participant after enrollment)***.
   1. Strongly agree
   2. Somewhat agree
   3. Neither agree nor disagree
   4. Somewhat disagree
   5. Strongly disagree
4. The STRIVE DDI screener has been helpful during ***discharge planning***.
   1. Strongly agree
   2. Somewhat agree
   3. Neither agree nor disagree
   4. Somewhat disagree
   5. Strongly disagree
5. Please rank the following elements of the STRIVE DDI screener in order of usefulness from ***1 (Most Useful) to 5 (Least Useful)***.
   1. Guidance on the screener home page (i.e., the screener search screen)
   2. Washout guidance for concomitant medications prior to first dose of study drug
   3. Start/restart guidance for concomitant medications
   4. Guidance on alternatives to prohibited medications
   5. Additional dosing guidance (i.e. dosing cap thresholds, reductions)

In Questions 6-10, please mark the most appropriate answer.

1. How clear has the **washout guidance** within the STRIVE DDI Screener for concomitant medications been when screening participants?
   1. Clear
   2. Unclear
      1. If "Unclear", Please list any specific medications or medication classes where washout guidance is unclear.
         1. Free text answer
2. How clear has the **start/restart guidance** been for concomitant medications ***started during the study drug course***?
   1. Clear
   2. Unclear
      1. If "Unclear", Please list any specific medications or medication classes where washout guidance is unclear.
         1. Free text answer
3. How clear has the **start/restart guidance** been for concomitant medications ***started at or after discharge***?
   1. Clear
   2. Unclear
      1. If "Unclear", Please list any specific medications or medication classes where washout guidance is unclear.
         1. Free text answer
4. In cases where there have been DDI concerns/issues **at screening**, how often have site pharmacists (either research or clinical) been involved with DDI concerns/issues at screening and when enrolling participants?
   1. Always
   2. Very often
   3. Sometimes
   4. Rarely
   5. Never
   6. Not applicable (there have been no DDI concerns/issues at screening)
5. When a **drug was not found in the screener**, how often was a pharmacist (either research or clinical) consulted to assist with questions regarding potential enrollment and/or clinical decisions?
   1. Always
   2. Very often
   3. Sometimes
   4. Rarely
   5. Never
   6. Not applicable (all needed drugs were found in the screener)
6. When a **drug was not found in the screener**, your site is most likely to do which of the following? ***Mark all that apply.***
   1. Utilize the drug resources available (e.g. DrugBank) to make enrollment/clinical decisions
   2. Consult pharmacy colleague (investigational or clinical)
   3. Await guidance from the central study team leadership
   4. Not enroll participant
   5. Unsure; this case has not happened yet
   6. Other:
      1. Free text comments
7. Please rate the **frequency of use** when screening a participant for each of the following STRIVE DDI trial materials:

|  | Always Used | Sometimes Used | Rarely Used | Never Used |
| --- | --- | --- | --- | --- |
| DDI Screener (link to screener) |  |  |  |  |
| DDI quick reference ***(Side 1: Permitted/Prohibited Medication List)***  (link to quick reference) |  |  |  |  |
| DDI quick reference ***(Side 2: Emergency Use Medication List)***  (link to quick reference) |  |  |  |  |
| Protocol Instruction Materials (i.e. PIM)  (link to PIM) |  |  |  |  |

1. Has your site referenced the STRIVE DDI screener within your electronic health record system (e.g. link to screener, best practice alerts, etc)?
   1. Yes
      1. If yes, then "How has your site integrated the STRIVE DDI screener into your electronic health record?" ***Mark all that apply.***
         1. Embedded link to STRIVE DDI screener into the electronic health record and/or order entry
         2. Best practice alerts for certain medications known to have DDI potential with S-217622/placebo
         3. Other:
            1. Free text comments
   2. No
   3. Not sure
2. In what way(s) does your site access the STRIVE DDI screener? ***Mark all that apply.***
   1. Electronic access on phone/tablet
   2. Electronic access on desktop/laptop
   3. Other:
      1. Free text comments
   4. We do not use the STRIVE DDI screener
3. If you have other feedback or comments pertaining to the STRIVE DDI screener, please describe below.
