# Supplementary material for: Implementation of an Online Drug–Drug Interaction Screener for the STRIVE Ensitrelvir Trial for COVID-19
Source: Open Forum Infect Dis. 2025 Jun 11;12(7):ofaf327. doi: 10.1093/ofid/ofaf327 (PMC12207740; doi:10.1093/ofid/ofaf327)
Supplement: ofaf327_Supplementary_Data [file ofaf327_supplementary_data.zip › Supplementary STRIVE Tables.docx]

|  | Medication Metabolism Pathway | | | |
| --- | --- | --- | --- | --- |
| Metabolism of the concomitant  medication: | Sensitive CYP3A substrate with narrow therapeutic index | Sensitive CYP3A substrates without narrow therapeutic index, or moderate-sensitive CYP3A substrate | Sensitive substrate of P-gp, BCRP, or  OAT3 | Strong or moderate CYP3A inducer; or strong CYP3A inhibitor |
| Timeframe for starting/ restarting the concomitant medication: | Study Day 29 | Study Day 15 | Study Day 5 | Study Day 5* |
| * Unless inducer or inhibitor is also a CYP3A substrate, then most conservative approach used   - Day 0 is the day of ESV randomization (i.e., first day of ESV/placebo treatment) - Most conservative timeline used if medication is substrate of any combination that includes CYP3A   Due to the long half-life of ESV (~48 hours) and persistent CYP3A inhibition following discontinuation, a slight DDI for CYP3A substrates persists even after Study Day 15. | | | | |

Supplementary Table 1. Start/Restart Timeline For Interacting Medications

Definitions: CYP, cytochrome P450; P-gp, P-glycoprotein; BCRP, breast cancer resistance protein; OAT-3, organic anion transporter-3.

| n (%) | Sites With Enrolled Participants (n=52)* |
| --- | --- |
| Total participants screened  0-9  10-50  51-100  101-500  >500 | 7 (13.5)  13 (25.0)  9 (17.3)  18 (34.6)  5 (9.6) |
| Total participants enrolled  1-5  6-10  11-15  >15 | 37 (71.2)  9 (17.3)  2 (3.8)  4 (7.7) |
| Total number of clinical trials as study site participant  0-1  2-3  4-5  >5  Unsure | 0 (0.0)  6 (11.5)  8 (15.4)  37 (71.2)  1 (1.9) |
| Dedicated pharmacist on site for DDI consultation  No  Yes  Unsure | 13 (25.0)  37 (71.2)  2 (3.8) |

Supplementary Table 2. DDI Screener Site Survey Characteristics.

*Represents sites who responded to the DDI screener survey and had enrolled ≥1 participant.
