# Supplementary figures and images for: Implementation of an Online Drug–Drug Interaction Screener for the STRIVE Ensitrelvir Trial for COVID-19
Source: Open Forum Infect Dis. 2025 Jun 11;12(7):ofaf327. doi: 10.1093/ofid/ofaf327 (PMC12207740; doi:10.1093/ofid/ofaf327)

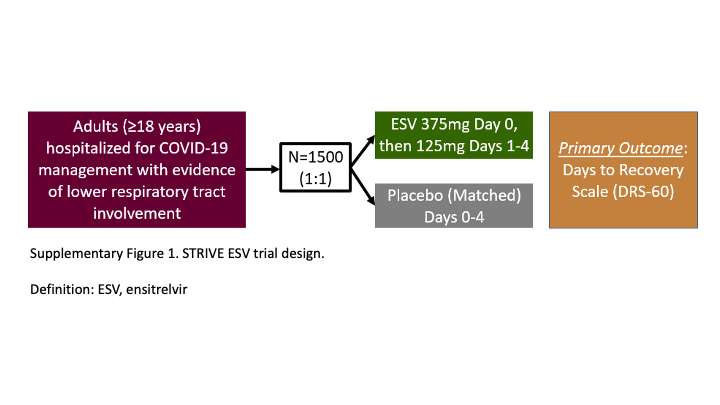

Supplement: ofaf327_Supplementary_Data [file ofaf327_supplementary_data.zip › SupFig1.STRIVE_Design.tiff]

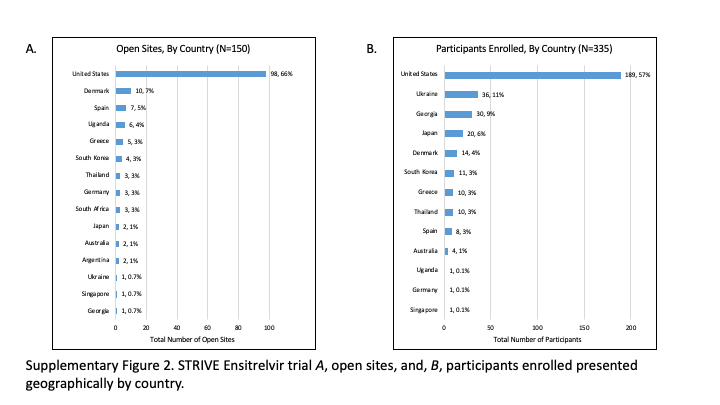

Supplement: ofaf327_Supplementary_Data [file ofaf327_supplementary_data.zip › SupFig2.STRIVE_Sites.tiff]

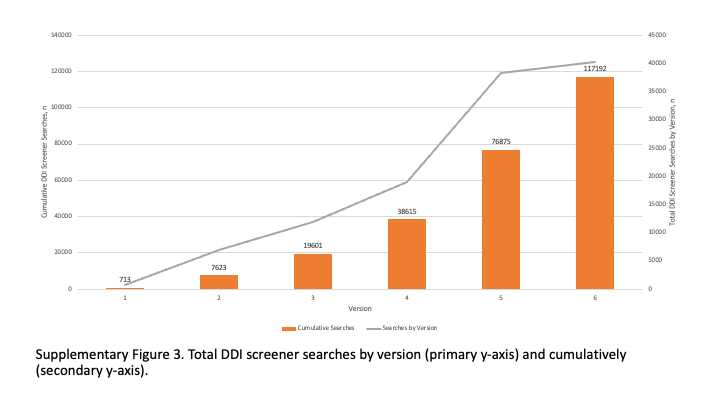

Supplement: ofaf327_Supplementary_Data [file ofaf327_supplementary_data.zip › SupFig3.ScreenerSearches.tiff]

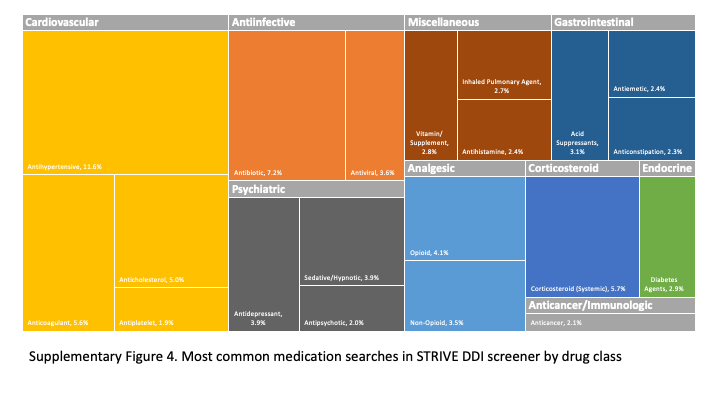

Supplement: ofaf327_Supplementary_Data [file ofaf327_supplementary_data.zip › SupFig4.TopMedicationsClass.tiff]

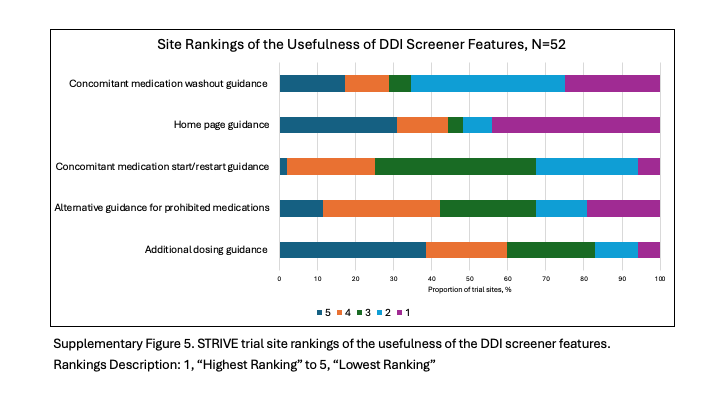

Supplement: ofaf327_Supplementary_Data [file ofaf327_supplementary_data.zip › SupFig5.ScreenerRankings.tiff]

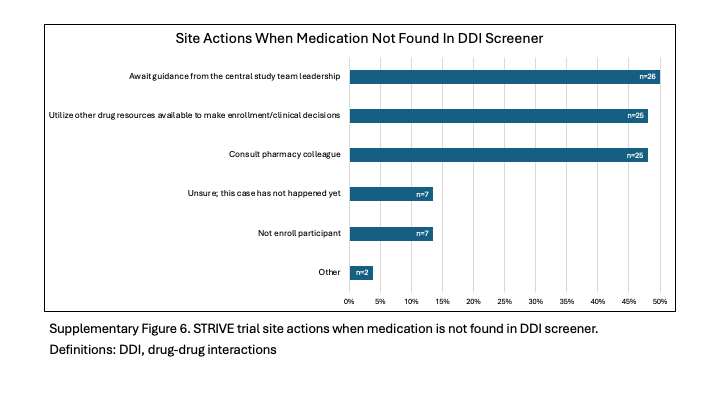

Supplement: ofaf327_Supplementary_Data [file ofaf327_supplementary_data.zip › SupFig6.MedNotFound.tiff]

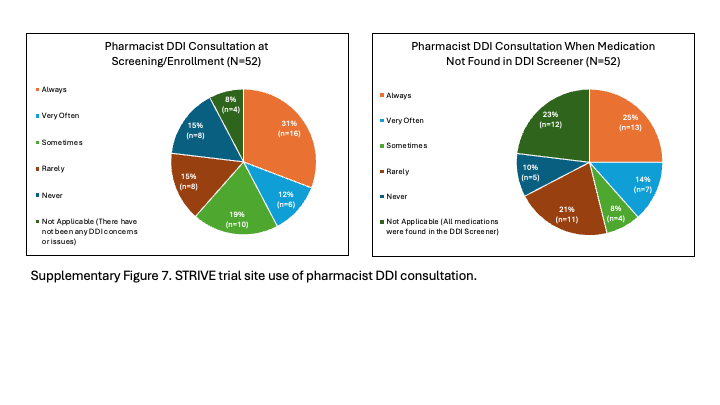

Supplement: ofaf327_Supplementary_Data [file ofaf327_supplementary_data.zip › SupFig7.PharmDConsultation.tiff]

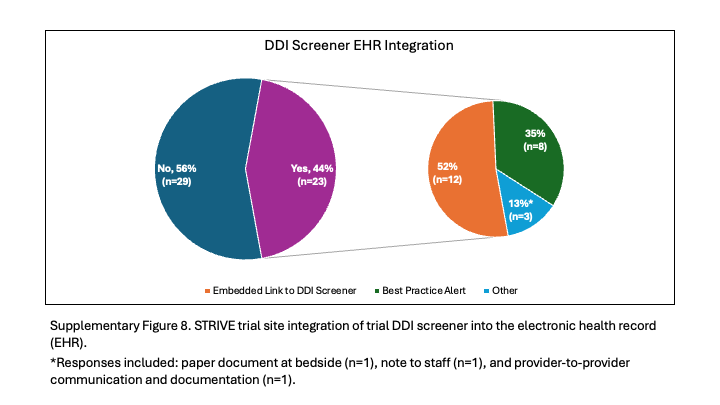

Supplement: ofaf327_Supplementary_Data [file ofaf327_supplementary_data.zip › SupFig8.EHR.tiff]
